# Design of a Compact Tuning Fork-Shaped Notched Ultrawideband Antenna for Wireless Communication Application

**DOI:** 10.1155/2014/874241

**Published:** 2014-03-02

**Authors:** M. N. Shakib, M. Moghavvemi, W. N. L. Mahadi

**Affiliations:** ^1^Department of Electrical Engineering, Faculty of Engineering, University of Malaya, 50603 Kuala Lumpur, Malaysia; ^2^Center of Research in Applied Electronics (CRAE), Faculty of Engineering, University of Malaya, 50603 Kuala Lumpur, Malaysia; ^3^Faculty of Electrical and Computer Engineering, University of Tehran, Tehran, Iran

## Abstract

A new compact planar notched ultrawideband (UWB) antenna is designed for wireless communication application. The proposed antenna has a compact size of 0.182*λ* × 0.228*λ* × 0.018*λ* where *λ* is the wavelength of the lowest operating frequency. The antenna is comprised of rectangular radiating patch, ground plane, and an arc-shaped strip in between radiating patch and feed line. By introducing a new Tuning Fork-shaped notch in the radiating plane, a stopband is obtained. The antenna is tested and measured. The measured result indicated that fabricated antenna has achieved a wide bandwidth of 4.33–13.8 GHz (at −10 dB return loss) with a rejection frequency band of 5.28–6.97 GHz (WiMAX, WLAN, and C-band). The effects of the parameters of the antenna are discussed. The experiment results demonstrate that the proposed antenna can well meet the requirement for the UWB communication in spite of its compactness and small size.

## 1. Introduction

With the drastic increase of market demand as well as rapid development of wireless communication, low power and high data rate communication systems have been becoming the key technologies in wireless communications. In this regard, the ultrawideband (UWB) technology is considered as latest and most popular short range wireless communication technology [1]. According to the Federal Communications Commission (FCC), a frequency band of 3.1–10.6 GHz with maximum radiated power of −41.3 dBm/MHz is considered for the UWB communication applications [2–4]. Recently, an enormous attention has taken place for designing UWB antenna due to its attractive characteristics of low power, light weight, high data rate capability, and easiness to be integrated with other devices. However, it is a challenging task to design and optimize an UWB antenna. Again, microstrip or planar antennas [5–8] present appealing physical features, such as low profile, smallness in size, conformability, easiness to be integrated with other devices, and low cost. Owing to these attractive characteristics, these antennas have gained attention to broaden the impedance bandwidth and use in the UWB system [9]. However, several narrowband communication systems such as WLAN IEEE 802.11a (5.725–5.825 GHz), WiMAX IEEE 802.16 (5.25–5.85 GHz), C-band (5.47–5.725 GHz or 5.725–5.875 GHz), and Extended C-Band (6.425–6.725 GHz) have been used for different applications. The UWB systems will cause interference with these existing narrowband communication systems. Therefore, it is desirable to filter the potential interference. For reducing or avoiding the potential interference between UWB systems and the narrow systems, the bandstop filters/notch should be applied in the antenna.

Various types of band notch techniques have been studied [9–11] such as using H-shaped conductor-backed plane [12], modifying two U-shaped slots on the patch [13], inserting two rod-shaped parasitic structures [14], using spurlines [15], embedding resonant cell in the microstrip feed-line [16], utilizing a small resonant patch [17], and using an MAM and genetic algorithm [18]. However, these antennas have either large radiator or are large in size. Printed or planar antennas with notch function have recently been studied in [19–23]. These antennas achieve notch band characteristics by integrating different types of narrow slots with the planar radiator. Recently, U-shaped or H-shaped slots embedded with radiating plate are introduced to obtain notched band frequencies in [21–23]. Again, by adding independent controllable strips in the radiating plate, a band notch characteristic is realized in [24]. More recently, a compact elliptical slot antenna [25] and a rectangular slot antenna with diamond patch [26] are developed to achieve notched band frequency with maintaining wideband characteristics. However, these antennas require a large radiator to operate in the desire frequencies.

In this paper, a new planar Tuning Fork-shaped notched compact antenna is presented for notched UWB operating characteristics. The bandwidth of the proposed antenna is increased by using rectangular radiating patch and an arc-shaped strip in between radiating patch and feed line. In addition, the proposed antenna is designed to have a rejection frequency band by introducing a new Tuning Fork-shaped notch in the radiating plane. This design has a compact dimension of 0.182*λ* × 0.228*λ* × 0.018*λ* which gives a low profile antenna to cover the UWB band with band rejection capability. In addition, the proposed antenna is compact compared to the designs recently reported in [19–26].

## 2. Antenna Design and Concept

**Figure 1:**
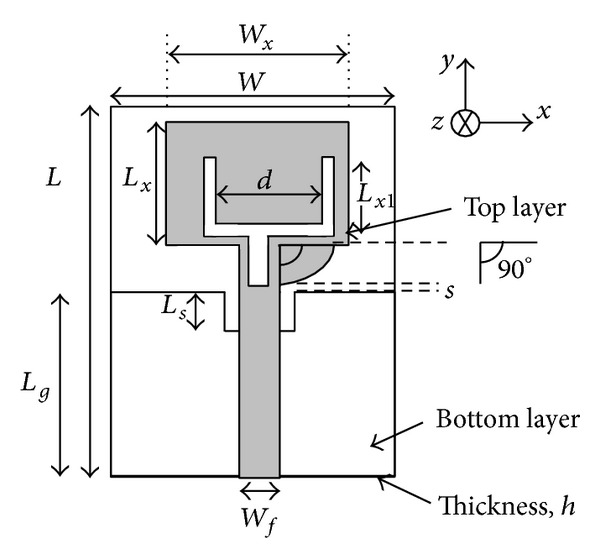
Geometry of the proposed notched antenna.

**Figure 2:**
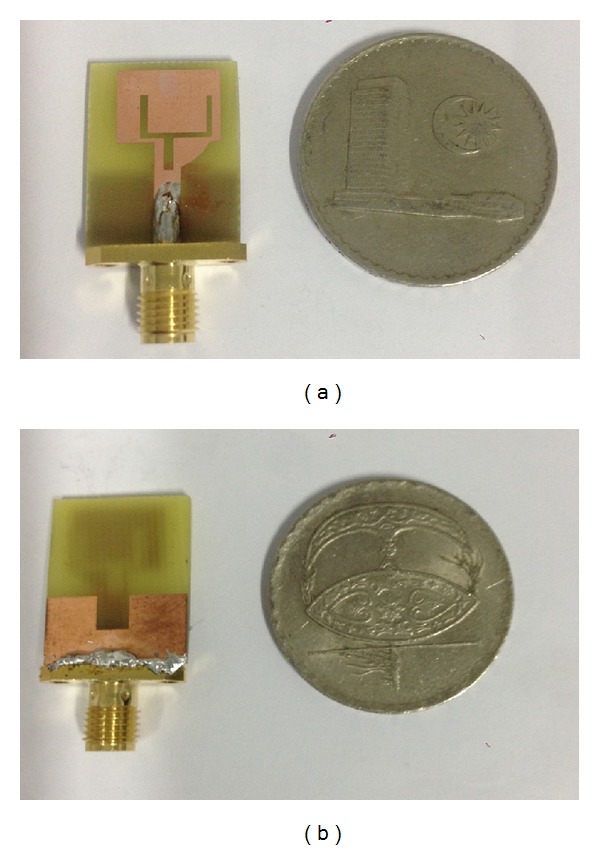
Fabrication of the proposed notched antenna. (a) Front view; (b) back view.

The geometries of the proposed notched ultrawideband antenna are shown in Figure 1. The antenna consists of radiating patch, notch ground plane, arc-shaped strip, and Tuning Fork-shaped notch. An FR4 substrate with relative permittivity of 4.4, a loss tangent of 0.002, and a thickness of 1.6 mm is used in antenna design. A transmission line with width of 2.8 mm is fed to match the 50 Ω characteristic impedance. In this design, on the front surface of the substrate, an arc-shaped strip connected from microstrip feed line to radiating patch is proposed. This arc-shaped strip acts as an impedance matching element to control the impedance bandwidth of the proposed antenna by creating additional surface current paths in the antenna. Therefore, the excited current shifts its upper resonances which results in wider impedance bandwidth at the higher frequency band. Furthermore, on the back surface of the substrate, a ground plane is placed and a rectangular notch/cut is loaded at the feeding point in the ground plane [27]. Therefore, by carefully adjusting the parameters of the notch, a better impedance match is observed. This leads to achieving a wider impedance bandwidth in the proposed design. In this design, a new Tuning Fork-shaped notch slot is introduced on the radiating patch and feed line. This notch stopped the band and controlled the current flow on the radiator. Thus, a band rejection characteristic is achieved. It clearly indicates that the rejection band of WiMAX, WLAN, and C-band is achieved by inserting Tuning Fork-shaped notch slot with appropriate length. At the notch frequency, a concentrated current is observed at the edges of the Tuning Fork-shaped notch slot. Hence, this leads to the desired high attenuation near the notch frequency. The fabrication of the proposed notched antenna is shown in Figure 2 where the antenna dimension is 0.182*λ* × 0.228*λ* × 0.018*λ* mm^3^. The parameters of the proposed antenna are summarized in Table 1.

## 3. Result and Discussions

**Figure 3:**
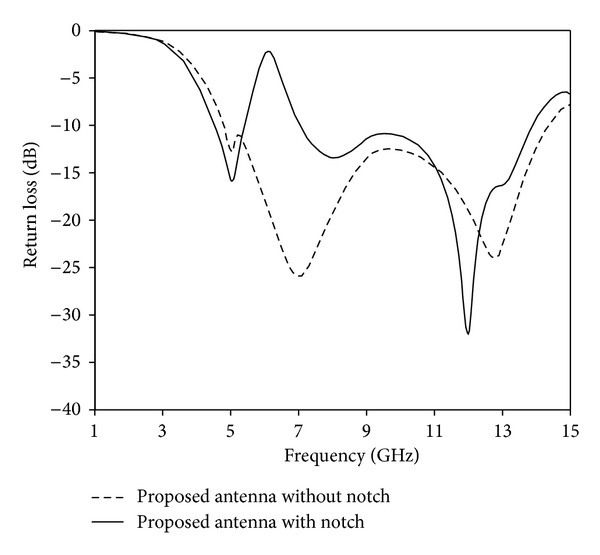
Simulated return loss of the proposed antenna with notch and without notch.

**Figure 4:**
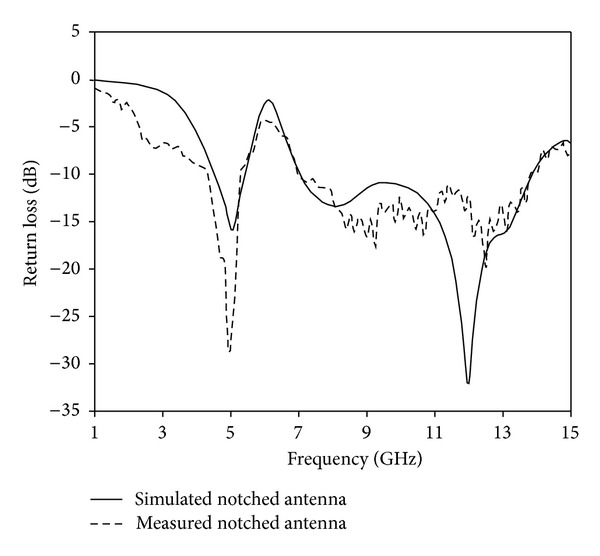
Measured and simulated return loss of the proposed notched antenna.

The analysis and performance of the proposed antenna are optimized by using HFSS software.  Figure 3 shows the simulated return loss performance of the proposed antenna with and without Tuning Fork-shaped notch. It can be observed that the proposed antenna without Tuning Fork-shaped notch achieves a bandwidth of 4.81 to 14.4 GHz and no stopband occurs. Again, with notch, the proposed antenna produces a stopband from 5.41 to 7.09 GHz.  Figure 4 shows the simulated and measured antenna with Tuning Fork-shaped notch. The proposed notched antenna is measured with a Rohde & Schwarz ZVA24 vector network analyzer. The measured result indicates that, at −10 dB return loss, the UWB antenna covers the bandwidth of 4.33–13.8 GHz with a band notch of 5.28–6.97 GHz. The small differences between the simulated and measured values may be due to the errors of the manufactured antenna. The proposed notched antenna characteristics show that by using a new Tuning Fork-shaped notch in the radiating patch, a band notch function is achieved which can cover the WLAN, 5.5 GHz WiMAX, and C-bands. Moreover, the proposed notched antenna is compact in size compared to the designs recently reported in [19–26].

**Figure 5:**
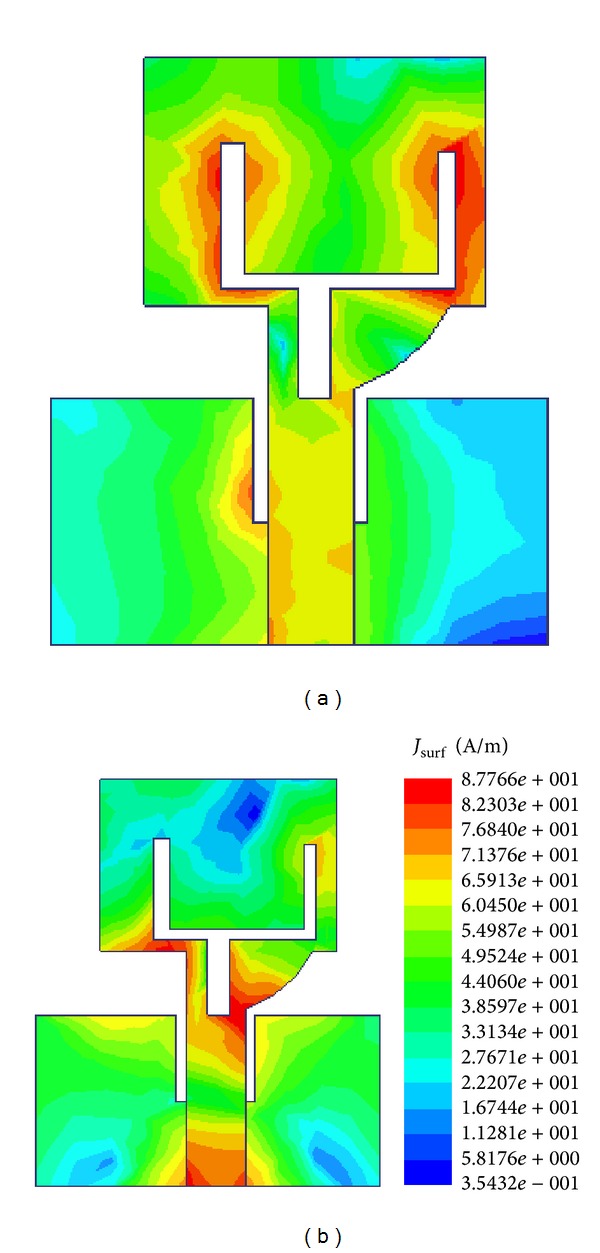
Current distribution of the proposed notched antenna. (a) 6 GHz; (b) 9 GHz.

**Figure 6:**
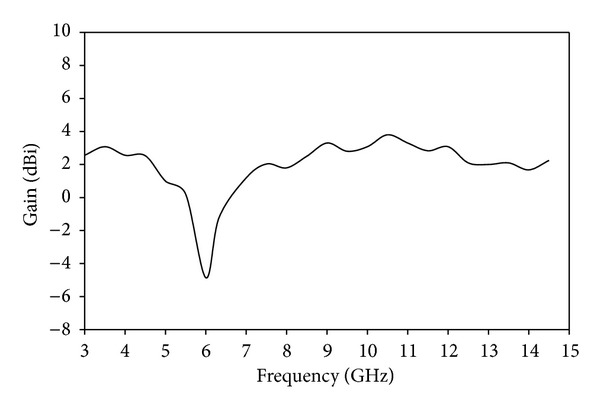
Measured gain of the proposed notched antenna.

**Figure 7:**
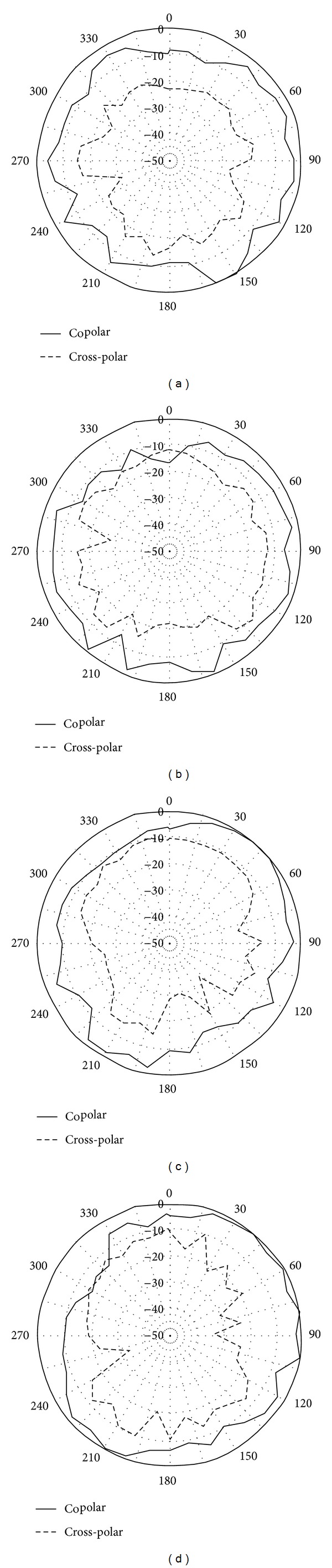
Measured radiation pattern of the antenna. (a) *xz*-plane at 5 GHz, (b) *yz*-plane at 5 GHz, (c) *xz*-plane at 9 GHz, and (d) *yz*-plane at 9 GHz.

Figure 5 illustrates the simulated current distributions on the notched antenna at 6 GHz and 9 GHz. At 6 GHz, it is observed that the majority of the electric currents are concentrated around the arm of the Tuning Fork-shaped notch. Thus, the Tuning Fork-shaped notch acts as good resonator at the corresponding rejection frequencies which creates impedance mismatching at the desired notched band. At 9 GHz, it is observed that the concentrated current flows basically on the feed line of the antenna. Thus, no band rejection is observed at 9 GHz.  Figure 6 shows the measured gain of the proposed antenna at different operating frequencies. A stable gain is obtained throughout the operation band except the notched frequency.  Figure 7 shows the measured radiation pattern of the proposed antenna. In the design, it can be seen that the antenna gives quasi-omni-directional characteristics.

**Figure 8:**
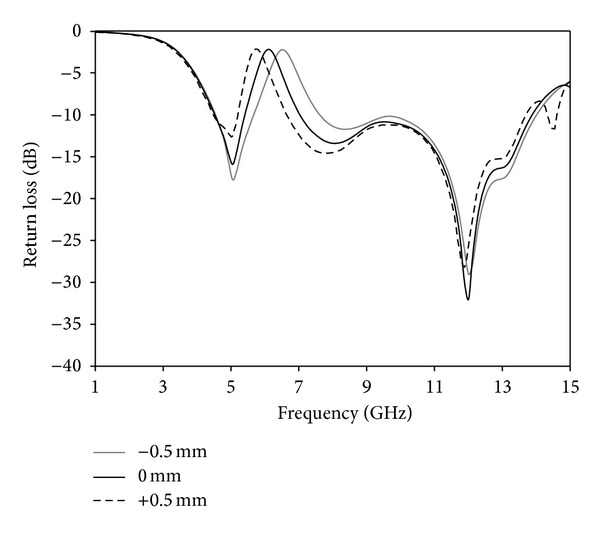
Effects on return loss with changing arm length.

**Figure 9:**
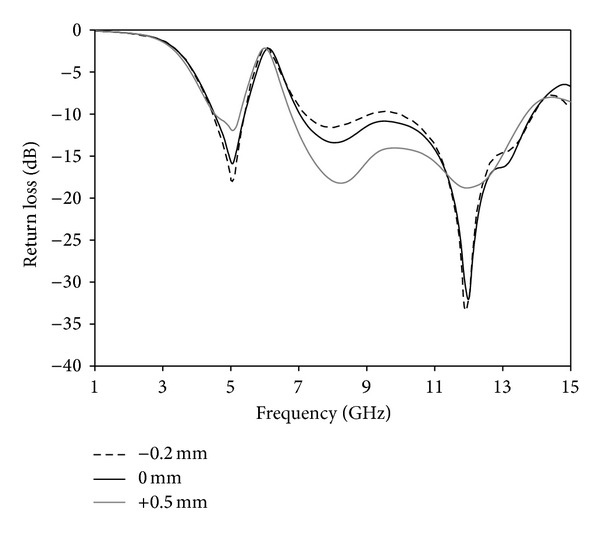
Effects on return loss with changing *s* parameter.

**Figure 10:**
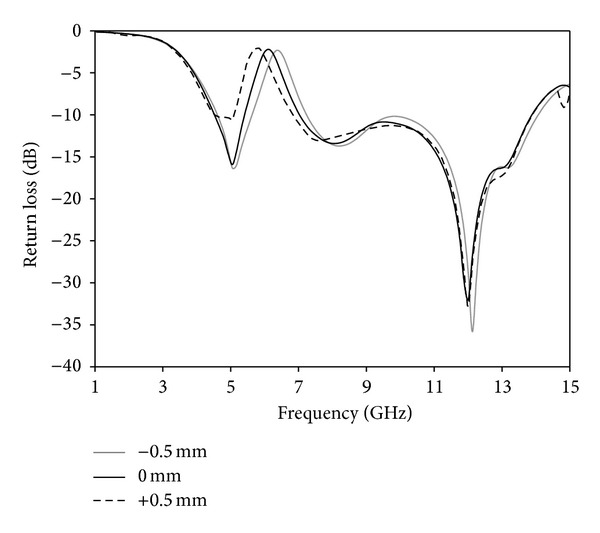
Effects on return loss with changing *d* parameter.

A parametric study is carried out to investigate the performance of the antenna. During the investigation, one parameter is changed while other parameters are fixed.  Figure 8 shows the effects of the various lengths of arm (*L*_*x*1_) of Tuning Fork-shaped notch on the return loss versus frequency. By increasing or decreasing, it has effect on the rejected frequency. By increasing the arm length, a frequency shift of almost 0.12 GHz occurs at the lower rejected band. Hence, an optimal value of the arm is chosen as 4.5 mm. An impedance matching due to changes of the *s* parameter is shown in Figure 9. Increasing *s* parameter experiences low matching at the lower and upper frequency band and it reduces the upper edge frequency, resulting in a reduction in the bandwidth. Again, decreasing *s* parameter affects matching around 6.8–11 GHz due to a decrease in the resistance. Hence, *s* = 0.2 mm is chosen as optimized parameter.  Figure 10 shows the effect of the *d* on the return loss. The distance between the arms of the Tuning Fork-shaped notch has an effect on the rejected frequency band. By increasing or decreasing the *d*, a frequency shift of almost 0.2 GHz occurs at the upper rejected band. Hence, an optimal value of *d* is chosen as 5.5 mm.

**Figure 11:**
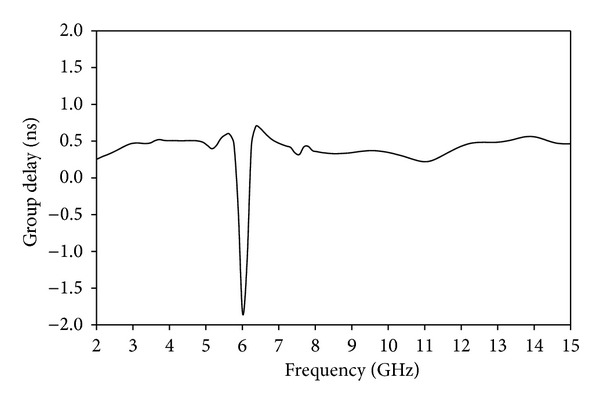
Group delay of the proposed notched antenna.

**Figure 12:**
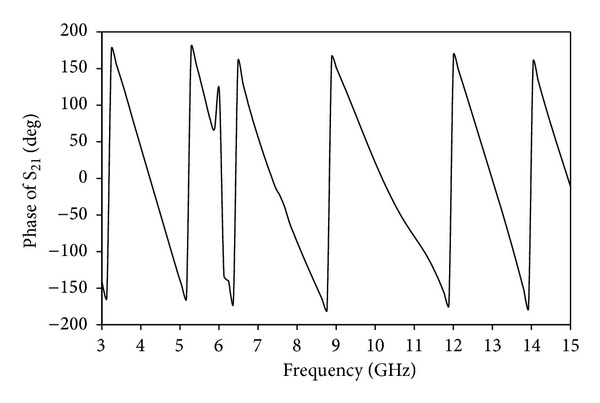
Phase of S_21_ of the proposed notched antenna.

Group delay is a significant parameter in UWB communication to signify the degree of pulse signal distortion. By representing the transmitting and receiving identical UWB antennas, the phase linearity and group delay at the operation band can be observed. To achieve a good linearity of the phase, the antenna should be able to transmit the signal pulse with minimum distortion. As shown from Figure 11, the group delay variation is less than 0.48 ns over the entire operating band except notched band. This indicates that the pulse transmitted or received by the antenna will not distort seriously. And, the group delay variation in the band notch frequency is higher which is expected from the proposed band notched antenna. Thus, these characteristics show that the proposed antenna is suitable to use for modern UWB wireless communication systems.  Figure 12 shows the Phase of S_21_ for the side-to-side scenario. The plot explains a linear variation of phase in the total operating band except notched band.

## 4. Conclusion

In this paper, a compact planar notched UWB antenna is designed and discussed for ultrawideband operation. In the design, a rectangular radiating patch, a notch ground plane, and an arc-shaped strip are used to achieve wider impedance bandwidth. By using the proposed techniques with Tuning Fork-shaped notch, the antenna achieves a wide bandwidth of 4.33–13.8 GHz with a rejection frequency band of 5.28–6.97 GHz (WiMAX, WLAN, and C-band). The notch in the design removes the unwanted frequency bands while maintaining an ultrawideband bandwidth performance. The antenna has a compact size of 0.182*λ* × 0.228*λ* × 0.018*λ*. The characteristics of compact size, low profile, and simple structure make the proposed antenna suitable to use for the UWB wireless communication applications.

## Figures and Tables

**Table 1:** Parameters of the proposed notched antenna.

Parameter	(mm)
*W*	16
*W*_*f*_	2.8
*W*_*x*_	11
*h*	1.6
*L*	20
*L*_*x*_	8
*d*	5.5
*L*_*x*1_	4.5
*s*	0.2
*L*_*g*_	8
*L*_*s*_	4
